# Phase II trial of Modified Vaccinia Ankara (MVA) virus expressing 5T4 and high dose Interleukin-2 (IL-2) in patients with metastatic renal cell carcinoma

**DOI:** 10.1186/1479-5876-7-2

**Published:** 2009-01-07

**Authors:** Howard L Kaufman, Bret Taback, William Sherman, Dae Won Kim, William H Shingler, Dorota Moroziewicz, Gail DeRaffele, Josephine Mitcham, Miles W Carroll, Richard Harrop, Stuart Naylor, Seunghee Kim-Schulze

**Affiliations:** 1 Tumor Immunology Laboratory, Division of Surgical Oncology, Columbia University, New York, NY, USA; 2 Oxford BioMedica U.K. Ltd., Oxford, UK; 3 R2D Ltd, Wantage, UK

## Abstract

**Background:**

Interleukin-2 (IL-2) induces durable objective responses in a small cohort of patients with metastatic renal cell carcinoma (RCC) but the antigen(s) responsible for tumor rejection are not known. 5T4 is a non-secreted membrane glycoprotein expressed on clear cell and papillary RCCs. A modified vaccinia virus Ankara (MVA) encoding 5T4 was tested in combination with high-dose IL-2 to determine the safety, objective response rate and effect on humoral and cell-mediated immunity.

**Methods:**

25 patients with metastatic RCC who qualified for IL-2 were eligible and received three immunizations every three weeks followed by IL-2 (600,000 IU/kg) after the second and third vaccinations. Blood was collected for analysis of humoral, effector and regulatory T cell responses.

**Results:**

There were no serious vaccine-related adverse events. While no objective responses were observed, three patients (12%) were rendered disease-free after nephrectomy or resection of residual metastatic disease. Twelve patients (48%) had stable disease which was associated with improved median overall survival compared to patients with progressive disease (not reached vs. 28 months, p = 0.0261). All patients developed 5T4-specific antibody responses and 13 patients had an increase in 5T4-specific T cell responses. Although the baseline frequency of Tregs was elevated in all patients, those with stable disease showed a trend toward increased effector CD8+ T cells and a decrease in Tregs.

**Conclusion:**

**V**accination with MVA-5T4 did not improve objective response rates of IL-2 therapy but did result in stable disease associated with an increase in the ratio of 5T4-specific effector to regulatory T cells in selected patients.

**Trial registration number:**

ISRCTN83977250

## Background

Renal cell carcinoma (RCC) is the fifth most common cancer worldwide and five-year survival is 9% for those with metastatic disease. High-dose bolus interleukin-2 (IL-2) is associated with a consistent and durable objective response in 17% of patients with metastatic RCC and a 6–9% complete response rate [1-3]. The relatively low frequency of therapeutic responses and significant treatment-associated toxicities, however, has made IL-2 difficult to recommend for all patients. The objective response rate to IL-2 was improved in a melanoma clinical trial when combined with gp100 peptide vaccination resulting in a 42% objective response rate [4]. In contrast to melanoma where numerous T cell specific antigens have been defined, relatively few antigens have been described in RCC [5].

5T4 is a membrane glycoprotein expressed at high levels on placental trophoblast and also on a wide range of human carcinomas including clear cell and papillary RCC but rarely on normal tissue [6,7]. 5T4 overexpression on tumor cells has also been associated with metastatic spread and poor prognosis in cancer patients [8,9]. 5T4 is not released from the cell membrane and thus can mediate antibody-dependent cell-mediated cytotoxicity (ADCC). In addition, 5T4-transduced renal carcinoma cell lines can be recognized by human T cells *in vitro*, suggesting that 5T4 can induce cellular immunity as well. 5T4-transfected tumor cells display altered morphology and increased motility suggesting that 5T4 plays a role in tumor progression and invasion [10]. A recombinant modified vaccinia virus Ankara (MVA) encoding human 5T4 (MVA-5T4) was tested previously in a phase I clinical trial for patients with stage IV colorectal carcinoma [11]. Vaccinated patients demonstrated few adverse events and nearly all patients developed 5T4-specific antibody and T cell immune responses, which correlated with time to disease progression [11]. Thus, the expression of 5T4 in RCC, ability to generate 5T4-specific humoral and cell-mediated immunity and the role of 5T4 in tumor progression suggest this would be an ideal antigen for targeted immunotherapy in RCC. Hence, we sought to determine if vaccination with MVA-5T4 could improve the therapeutic responses observed with standard high-dose IL-2 in patients with metastatic RCC. In order to take advantage of IL-2 during the contraction phase of the immune response, we designed an exploratory trial in which an initial vaccination was administered alone and subsequent booster immunizations were supported by the addition of high-dose bolus IL-2.

## Methods

### Patients

This phase II trial was an open label study of MVA-5T4 vaccine in patients with metastatic clear cell or papillary RCC eligible for high-dose IL-2. A total of 25 patients were enrolled who met these criteria: Eastern Cooperative Oncology Group (ECOG) performance status of 0 to 1, life expectancy greater than six months, 18 years of age or older; able to provide written informed consent; able to comply with study procedures, hemoglobin > 10 g/dL, granulocyte count > 1500/mm3, lymphocyte count > 1000/mm3, platelet count > 100,000/mm3, serum creatinine < 2.5 mg/dL, total bilirubin < 1.5 × the normal upper limits, and AST, ALT, and alkaline phosphatase < 3 × the normal upper limit, or < 5 × the normal upper limit if due to liver metastases. The clinical protocol was approved by the Institutional Review Board.

### Vaccine preparation

5T4-MVA vaccine was produced by homologous recombination of human 5T4 cDNA into deletion region III of MVA under the control of the modified H5 promoter, as previously described [12]. Individual vials were stored in a secured, monitored, alarmed refrigerator at -80°C. A sterile syringe was used to inject 1 mL of solution subcutaneously in the deltoid region.

### Study design

A dose of 5 × 10^8 ^pfu (1 ml) MVA-5T4 was established as safe in a Phase I trial [11]. In this trial, the first dose was given by intramuscular injection alone and booster vaccination was given 3 weeks later, followed immediately by high dose IL-2 (600,000 IU/kg) given every 8 hours up to a maximum of 15 doses. Three weeks later patients received a third booster and second cycle of IL-2. All patients underwent re-staging CT scans two weeks later. Clinical responses were determined by RECIST criteria [13]. For patients without progression an additional two cycles of vaccine/IL-2 were given at three week intervals. Patients demonstrating benefit after completing two courses of IL-2 were allowed to continue vaccination every three months for up to one year. In order to monitor the immune responses prior-, during- and post-vaccinations, heparinized blood was collected and processed by centrifugation through Histopaque columns to isolate peripheral blood mononuclear cells (PBMC).

### Antibody responses

MVA- and 5T4-specific antibody titers were determined by ELISA as described previously [11]. All test plasma was compared against a pool of plasma taken from 50 healthy (vaccinia naïve) donors. Antibody titers were defined as the greatest dilution of plasma at which the mean optical density (O.D.) of the test plasma was ≥ 2 fold the mean O.D. of the negative control (normal human plasma) at the same dilution. A positive response was defined as a post-vaccination titer ≥ 2 fold of the baseline titer.

### T cell responses

The IFN-γ ELISPOT was used to monitor T cell responses, as previously described [14]. Briefly, frozen PBMCs were thawed and incubated in medium overnight at 37°C, 5% CO2 prior to use. ELISPOT plates (PVDF, Millipore) were coated with an anti-IFN-γ capture antibody (human IFN-γ ELISPOT kit, Mabtech). Following blocking, 2 × 10^5 ^PBMCs were added to each well and incubated overnight at 37°C, 5% CO_2 _with the appropriate antigens. For positive control CEF (CMV, EBV and Flu virus) 10 amino acid length peptides were used. Subsequently, spots were enumerated using an automated ELISPOT plate reader. The precursor frequency was calculated as the number of spot-forming units from wells containing PBMC and 5T4 overlapping peptides after subtraction of the background (PBMC alone) relative to the number of PBMC seeded per well. A positive ELISPOT response was reported if the mean spot forming units (SFU) per well in response to antigen was ≥ 3 fold the mean SFU/well in wells containing medium alone and the mean SFU/well in response to antigen was ≥ 10. A positive response was also required to demonstrate ≥ 2 fold increase after vaccination. Phenotypic characterization was done by four color flow cytometry analysis of PBMC using the following antibodies: CD4, CD8, CD25, CCR7, CD45RA, Foxp3, GITR, PD-1, IL-10, CD152, CD107a, granzyme B and perforin. Isotype matched controls were always included. The change of frequency for specific subset of cells during the post-vaccination period is calculated by subtracting the basal value of pre-vaccination time point. Flow cytometry was done using a FACSCalibur flow cytometer equipped with CellQuest Pro software. T cell function was tested by mixed lymphocyte proliferation assay, as previously described [15]. A total of 16 healthy donor PBMC were used as normal controls.

### Statistical analysis

Since this was an exploratory study, no formal power calculations were undertaken. The intention-to-treat population included all subjects enrolled in the study and the per-protocol population met all eligibility criteria and completed at least five vaccinations. All safety and efficacy analyses were carried out using the intention-to-treat (ITT) population and analysis of immune response was carried out in the per-protocol population. Descriptive statistics were analyzed using Student's *t*-test to assess differences between the different study groups with p < 0.05 considered significant. Correlations between variables were assessed with adjustments to other variables via linear models. Overall survival (OS) was calculated by the method of Kaplan-Meier, log rank test. OS was calculated from the first date of treatment to date of death, or last known date alive.

### Role of funding source

This work was supported by grants from Oxford Biomedica. The funding sources had no role in the study design, collection, analysis, or interpretation of the data, or in the writing of the report. They also had no access to the raw data. The corresponding author had full access to all data and the final responsibility to submit for publication.

## Results

### Patient characteristics

Twenty five patients were enrolled in the trial and included in the ITT population. One patient withdrew from the trial early due to relocation and one patient could not tolerate IL-2, leaving 23 patients in the per-protocol analysis. The mean age of the ITT population was 58.4 ± 10 years (range 44 – 77 years). 21 patients had clear cell carcinomas and 4 patients had papillary histology. Further characteristics are detailed in Table 1.

**Table 1:** Patient characteristics and treatments

Mean age	58.4 (range 44–77)			
			N = 25	**%**
			
Sex	Male		17	68
	Female		8	32

TNM Stage	T	X	4	16
		0	0	0
		1	4	10
		2	7	28
		3	7	28
		4	3	12
	
	N	X	0	0
		0	18	72
		1	0	0
		2	7	28
	
	M	0	0	0
		1	25	100

Histology	Clear cell	21	84	
	Papillary	4	16	

Sites of disease	Lung	16	64	
	Lymph node	9	36	
	Soft tissue	7	28	
	Bone	6	24	
	Kidney	5	20	
	Liver	5	20	
	Pancreas	2	8	
	Adrenal	1	4	

Prior Therapy	Nephrectomy	23	92	
	Chemotherapy	8	32	
	Immunotherapy	10	40	
	Radiation therapy	2	8	
	Cryoablation	1	4	
	Laser ablation	1	4	

**Treatment Characteristics**				

Vaccination	≤ 2	3	12	
	3–5	14	56	
	≥ 6	8	32	

No. of IL-2 Cycles	1	4	16	
	2	9	36	
	3	0	0	
	4	9	36	

### Treatment-related toxicity

Table 2 shows all adverse events; there were no serious adverse events related to the vaccine in the ITT population. The most frequent side effect related to vaccine administration was fever in 8 patients. Other toxicities were largely expected high-dose IL-2 related side effects (see Table 2).

**Table 2:** Adverse events related to vaccine and IL-2

Vaccine-related AEs		Maximum Grade	Patients	
		
System	Adverse Events		N = 25	%
Constitutional	Fever	1	8	32
	Pain at injection site	1	4	16
	Injection site reaction	1	3	12
	Myalgia	1	1	4
	Chills	1	1	4

IL-2-related AEs				

Cardiovascular	Cardiopulmonary arrest	4	1	4
	Elevated troponin	4	1	4
	Hypotension	4	9	36
	Ventricular tachycardia	3	1	4
	Acidosis	4	1	4

Electrolyte	Hyperglycemia	3	4	16
	Hypocalcemia	3	1	4
	Hyponatremia	3	11	44
	Hypophosphatemia	3	3	12

Gastro-intestinal	Ischemic bowel	4	1	4

Hematologic	Anemia	3	2	8
	Neutropenia	3	1	4
	Thrombocytopenia	3	4	16

Hepatic	Elevated transaminases	3	1	4
	Hyperbilirubinemia	3	3	12

Neurologic	Confusion	3	3	12
	Syncope	3	2	8

Pulmonary	Dyspnea	3	1	4

Renal	Elevated creatinine	3	22	88
	Oliguria	3	2	8

Systemic	Fatigue	3	1	4

### Humoral immune responses

MVA- and 5T4-specific antibody responses were monitored by ELISA at each sampling time point throughout the trial and expressed as a titer [see Additional file 1]. All patients showed an increase in MVA antibody titers following vaccination (range, 4000 to 128,000). One patient (#19) had detectable MVA-specific titers prior to the first vaccine and this increased further following vaccination. All patients also demonstrated 5T4-specific antibody titers ranging from 20 to 2560, which were evident after ≥ 2 vaccinations in most patients. Two patients (#13 and 23) had detectable 5T4-specific antibody titers prior to vaccination but showed an increase in post-immunization titers.

### Effector and regulatory T cell responses

**Figure 1:**
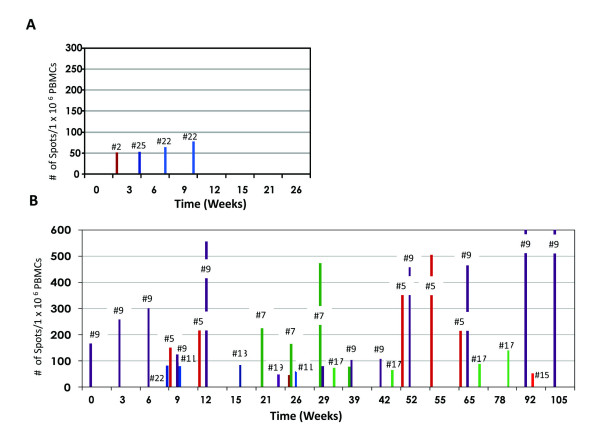
**5T4-specific T cell responses in patients with (A) progressive disease and (B) stable disease**.

5T4-specific CD8+ T cell responses were monitored by IFN-γ ELISPOT assay using overlapping 5T4 peptides and full-length protein. Before immunization only a single patient had a detectable T cell response (frequency 1:5,618). Following treatment 13 of 23 tested patients (57%) had detectable 5T4-specific CD8+ T cell responses with precursor frequencies ranging from 1:21,277 to 1:1,792 (Table 3). Only 3 of 11 (27%) patients with progressive disease exhibited an increase in T cell response compared to 10 of 12 patients (83%) with stable disease (Fig. 1). Positive T cell responses to MVA and a control CEF peptide pool were detected in all 23 evaluable patients (Table 4). The CEF-specific precursor frequencies were highly consistent throughout the study period. The mean frequency of MVA-specific T cells was decreased slightly from 1:615 PBMCs pre-vaccination (1.62%) to 1:945 PBMCs post-vaccination (0.105%).

**Table 3:** Antigen specific T cell responses

Patient Number	Peak 5T4 polyclonal precursor frequencies				ORR (month)
		
	Time point (week)	Peptides alone	Time Point (week)	Protein + Peptides	
1	-	< 1/200,000	-	< 1/200,000	PD
2	-	< 1/200,000	3	**1/19,231**	PD
3	-	< 1/200,000	-	< 1/200,000	PD
5	55	**1/12,048**	55	**1/1,701**	**Surgical CR(50+)**
6	26	**1/21,277**	26	**1/21,277**	**SD (13)**
7	29	**1/2,113**	29	**1/2,113**	**SD (9)**
8	-	< 1/200,000	-	< 1/200,000	PD
9	105	**1/993**	105	**1/993**	**Surgical CR(54+)**
10	-	< 1/200,000	-	< 1/200,000	PD
11	-	< 1/200,000	9	**1/12,500**	**SD (2)**
12	-	< 1/200,000	-	< 1/200,000	PD
13	15	**1/11,765**	15	**1/11,765**	**SD (1)**
14	-	< 1/200,000	-	< 1/200,000	PD
15	-	< 1/200,000	92	**1/19,231**	**SD (18)**
16	-	< 1/200,000	-	< 1/200,000	**SD (2)**
17	78	**1/7,042**	78	**1/4,132**	**SD (16)**
19	-	< 1/200,000	20	**1/20,833**	**SD (5)**
20	-	< 1/200,000	-	< 1/200,000	PD
21	-	< 1/200,000	-	< 1/200,000	PD
22	9	**1/12,821**	9	**1/12,821**	PD
23	-	< 1/200,000	-	< 1/200,000	**Surgical CR (13+)**
24	-	< 1/200,000	6	**1/12,048**	**SD (3.5)**
25	-	< 1/200,000	3	**1/18,868**	PD

The peak 5T4 specific responses detected at any time point to 5T4 peptides or 5T4 peptide plus protein. *; no detection of 5T4 responses. Positive responses are indicated as bold type.

**Table 4:** T cell responses to CEF and MVA antigens by IFN-γ ELISPOT

Patient Number	Antigen	Peak Ag Specific T cell Precursor Frequencies	
		
		Pre	Post
1	CEF	ND	1/1,299
	MVA	ND	1/11,364
2	CEF	ND	1/10,929
	MVA	ND	1/4,926
3	CEF	ND	ND
	MVA	1/18,182	1/10,341
5	CEF	ND	1/1,658
	MVA	ND	1/3,993
6	CEF	< 1/200,000	< 1/200,000
	MVA	1/4,411	1/2,629
7	CEF	ND	1/2,084
	MVA	ND	1/1,935
8	CEF	1/1,613	1/1,126
	MVA	< 1/200,000	1/1770
9	CEF	1/1,040	1/956
	MVA	1/5,263	1/1,452
10	CEF	< 1/200,000	< 1/200,000
	MVA	< 1/200,000	1/3,442
11	CEF	1/5,882	1/2,362
	MVA	1/3,030	1/2,135
12	CEF	ND	< 1/200,000
	MVA	< 1/200,000	1/45455
13	CEF	< 1/200,000	< 1/200,000
	MVA	< 1/200,000	1/29,630
14	CEF	1/631	1/619
	MVA	1/928	1/2,112
15	CEF	1/1,357	1/1,445
	MVA	1/3,731	1/2,901
16	CEF	1/2,070	1/2,316
	MVA	1/2,618	1/2,685
17	CEF	ND	1/10,216
	MVA	ND	1/5,405
19	CEF	1/1,543	1/2,335
	MVA	1/5,618	1/2,273
20	CEF	< 1/200,000	< 1/200,000
	MVA	1/868	1/984
21	CEF	< 1/200,000	< 1/200,000
	MVA	1/1,230	1/945
22	CEF	ND	1/1,789
	MVA	ND	1/1,988
23	CEF	1/629	1/1,056
	MVA	1/5,208	1/4,561
24	CEF	1/870	1/1,078
	MVA	1/1,923	1/23,256
25	CEF	1/1,538	1/1,161
	MVA	1/7,143	1/3,697

Abbreviation: ND, not detected

**Figure 2:**
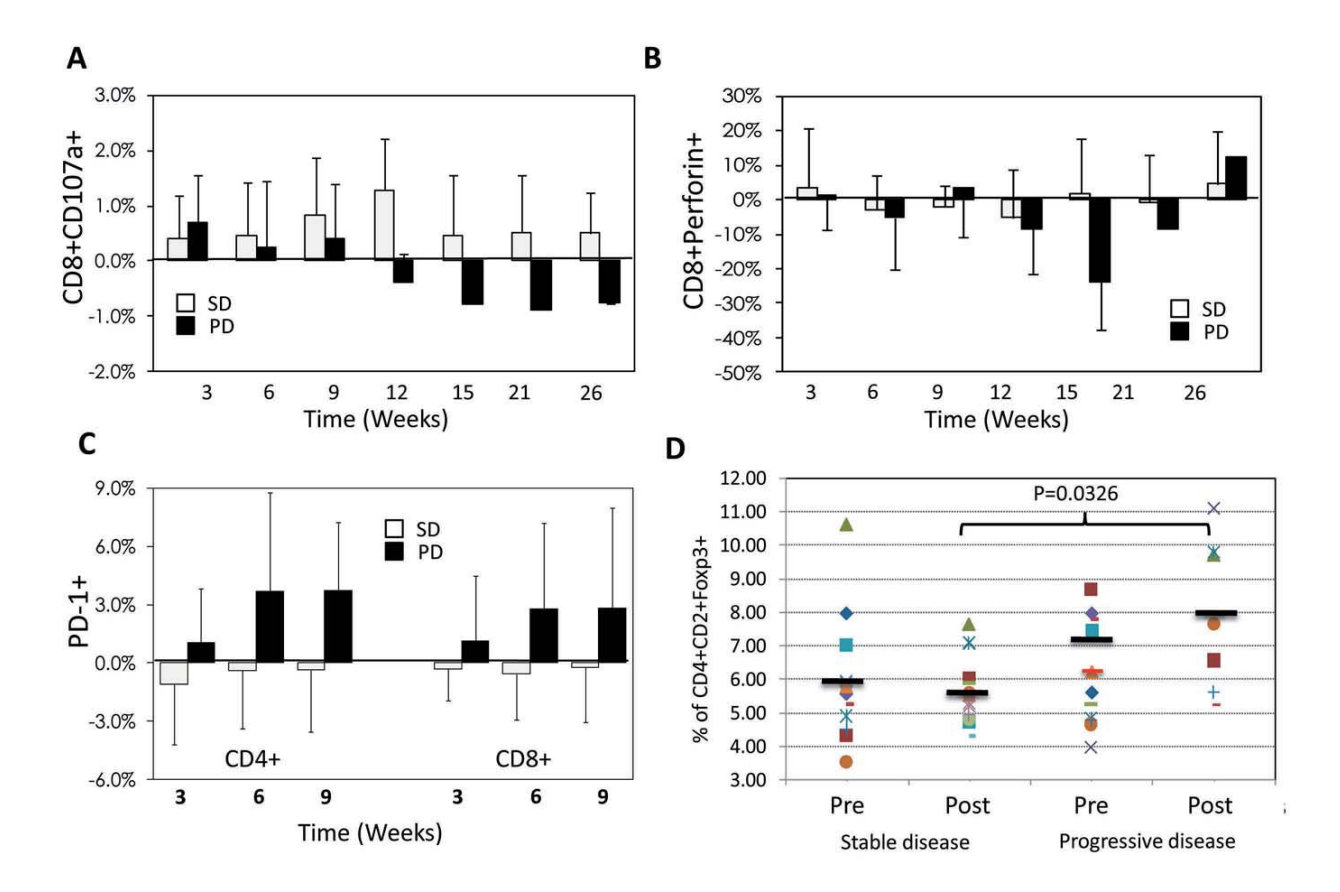
**Characterization of T cell responses**. (A) CD8+CD107a+ effector cells, (B) CD8+perforin+ effector cells, (C) PD-1+ T cells, (D) CD4+CD25+FoxP3+ Tregs before and after treatment.

CD8+ effector T cell response were also characterized by staining for T cell activation markers [16,17]. The mean frequency of CD8+CD107a+ T cells at baseline was 1.80% ± 0.95 and increased to 2.10% ± 0.64 after vaccination. Figure 2A shows that patients with stable disease had a significantly greater increase in CD8+CD107a+ T cells compared to those with progressive disease (1.50% ± 0.72 vs. 2.09% ± 0.30, p = 0.015). There was also a higher frequency of CD8+perforin+ T cells in RCC patients compared to normal healthy donors (27.58 vs. 15.25%, p = 0.020) and a trend towards decreasing CD8+perforin+ T cells in patients with progressive disease (Fig. 2B). In addition, there was a significant increase in PD-1 expressing CD4+ (p = 0.0329 at 3 weeks and p = 0.0281 at 9 weeks) and CD8+ T cells (p = 0.0373 at 3 weeks) in patients with progressive disease compared to stable patients (Fig. 2C).

**Figure 3:**
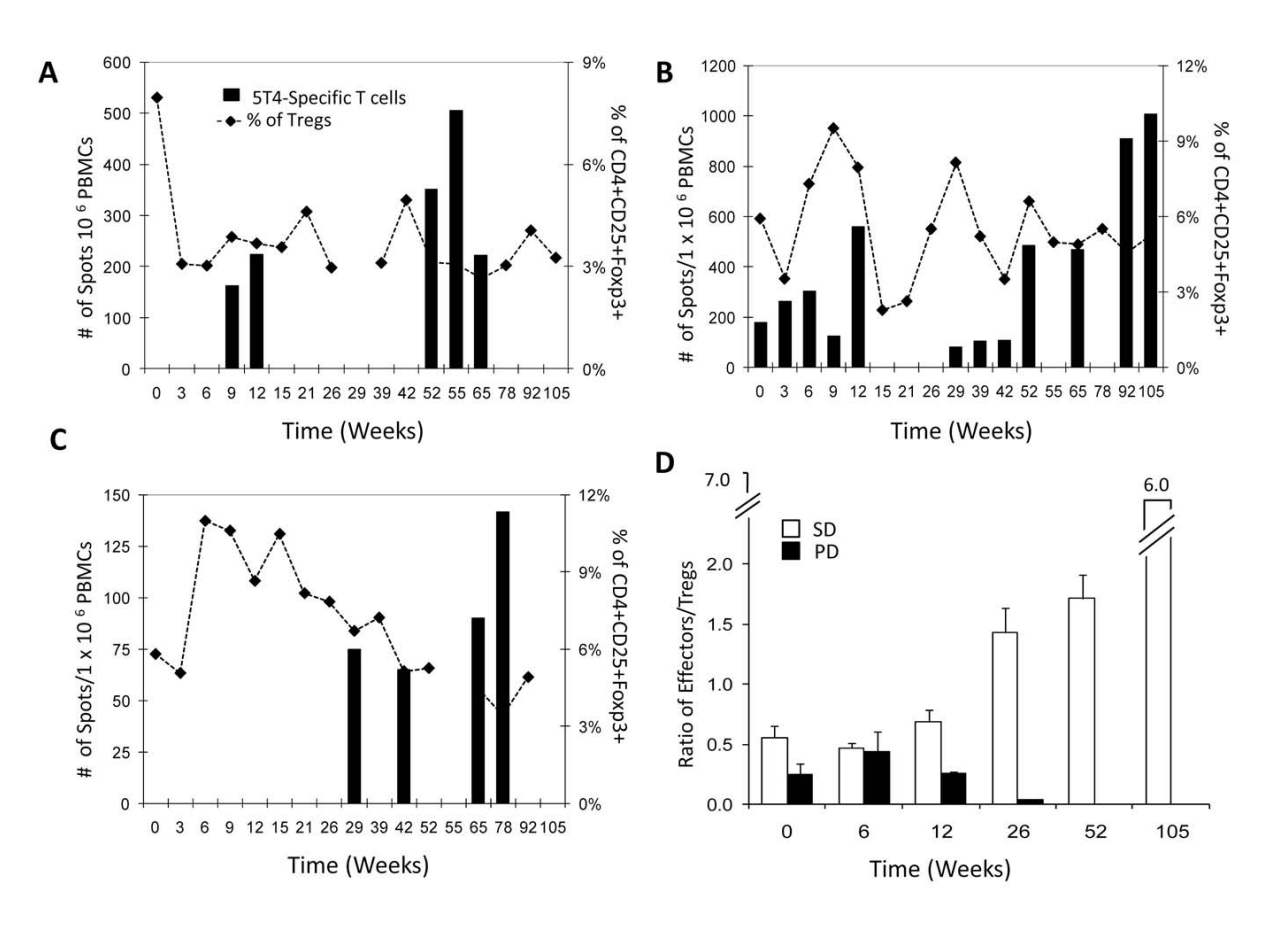
**Representative effector CD8+ T cell and Treg responses in 3 patients (A-C)**. effector/regulatory T cell ratio in all patients (D). SD, stable disease (open square), PD, progressive disease (closed square).

CD4+CD25+FoxP3+ Tregs were monitored by flow cytometry throughout the trial and functional suppression determined by co-culture proliferation assay. The mean frequency of Tregs in the per-protocol population at baseline was significantly higher than that detected in healthy donors (6.54% vs. 1.42%, p = 0.00002), although the degrees of suppression in proliferation assays was similar (p = 0.80) (data not shown). In patients with progressive disease, the mean Treg frequency was 7.03% (± 3.21) before treatment and increased to 8.00% (± 6.93) after treatment (Fig. 2D). In contrast, patients with stable disease had a mean Treg frequency of 5.93% (± 1.90) prior to treatment which decreased to 5.60% (± 2.43) by 15 weeks (Fig. 2D). The absolute number of Tregs was decreased by 50% in stable patients following treatment (p = 0.006). Fig. 3E–G shows the kinetics of effector CD8+ T cell responses and Treg frequency in three representative patients with stable disease. The effector/regulatory T cell ratio decreased in patients with progressive disease, whereas stable patients showed a dramatic increase which was maintained for up to 24 months (Fig. 3H).

### Clinical response

**Figure 4:**
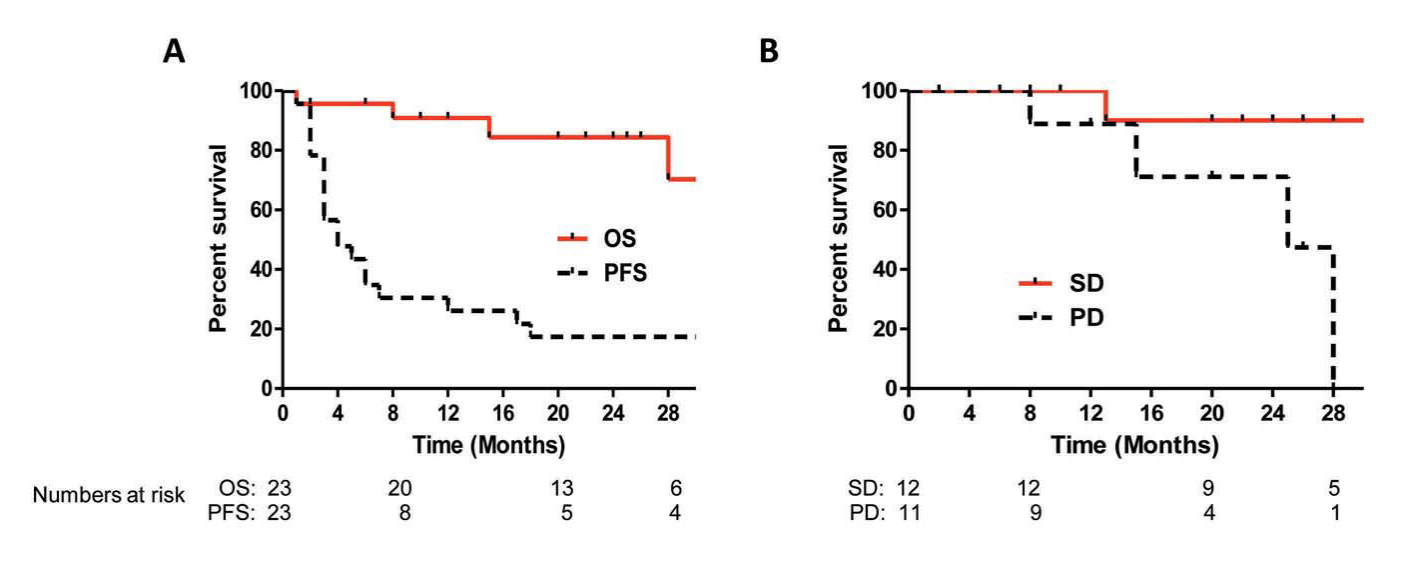
**Kaplan-Meier analysis of (A) overall (solid line) and progression-free (dashed line) survival of per-protocol patients treated with MVA-5T4 and IL-2**. (B) Overall survival of stable (solid line) and progressive (dashed line) disease patients. Numbers of patients at risk at 8, 20 and 28 months are shown below the graph.

There were no objective responses based on the first re-staging CT scans. Twelve of 23 (52%) per-protocol patients, however, had stable disease and went on to a second course of vaccination/IL-2. Three patients (13%) were rendered disease free through surgical resection; 2 patients had complete regression of all metastatic disease (lungs and bone) at initial follow-up and underwent nephrectomy of primary tumors, 1 patient had two intra-abdominal masses that regressed by < 20% but were surgically resected (pathology showed tumor with significant necrosis in one mass and no viable tumor in the other). The median progression-free survival of the per-protocol patients was 4.76 months and median overall survival has not yet been reached (Fig. 4A) at a median follow-up of 20 months.

Median overall survival of the 12 stable patients has not yet been reached (8–32 months) and was 28 months (2–28 months) for those with progressive disease (Fig. 4B, p = 0.0206).

## Discussion

This study established the safety and feasibility of combining vaccination with MVA expressing 5T4 and high-dose IL-2 in patients with metastatic RCC. The trial was initially designed to determine the impact of combination treatment on objective response rate since there is a well-defined, consistent response for IL-2 alone [1,2]. We did not, however, observe any objective responses by strict RECIST criteria although three patients were rendered disease free by additional surgery. The reasons for this outcome might relate to the study design in which we evaluated initial tumor responses two weeks after completing the first course of IL-2, selected in order to continue booster immunizations in a timely manner. Recent reports suggest that the kinetics of immunotherapy may require more time to mediate tumor regression in patients with established disease and, therefore, detection of tumor regression may be delayed [18,19]. This possibility is supported by patient #17, who continues to have a slow but steady regression of tumor over a 24 month period. Thus, our decision to scan at two weeks might have prevented some patients with stable disease from becoming objective responders. The trial was also biased by the early surgical intervention in three patients who were rendered disease free prior to further follow-up imaging. Two of these patients had complete regression of metastatic disease but had large primary renal tumors in place. Primary tumors are known to be more resistant to immunotherapy and often require nephrectomy before or after treatment to optimize response [20]. We also included four patients with papillary histology in the trial since these tumors express 5T4, but these tumor are also more resistant to IL-2, which may have influenced our results [21].

MVA-5T4 vaccine and high-dose IL-2 elicited 5T4-specific humoral and cell-mediated immunity. All patients developed an increase in 5T4 antibody titers after vaccination, consistent with previous clinical trials in patients with metastatic colorectal and hormone-refractory prostate cancer [11,22]. While the pattern of antibody response in our patients was similar to that observed in previous studies, the magnitude of the response was higher in this trial (mean 220, maximum titer 2560) compared to colorectal cancer patients treated with MVA-5T4 and chemotherapy (mean 76, maximum titer 1280) [14]. We also observed the induction of 5T4-specific CD8+ T cell responses in 57% (13/23) of vaccinated patients and this compares favorably to previous trials [11,14]. The induction of humoral and T cell immunity in this trial might relate to the underlying tumor histology, since RCC is known to be more immunogenic than other tumors [23,24] or could be due to the adjuvant effects of high-dose IL-2. We further characterized the effector CD8+ T cells in whole PBMC and found that there was an increase in CD107a, a marker of degranulation and cytotoxic function [16,17]. These cells remained elevated in patients with stable disease but began to decrease at 12 weeks in patients with progressive disease. We saw a similar trend in CD8+perforin+ T cells although this was only significant at 15 weeks. We also found that PD-1 expression, a pan T cell co-inhibitory receptor, was significantly elevated in both CD4+ and CD8+ T cells in patients with progressive disease [25-27]. These data suggest that the loss of effector CD8+ T cells or decreased effector function is associated with tumor progression.

Since Tregs may suppress tumor rejection by effector T cells and because IL-2 can promote Treg activity, we evaluated the frequency and functional activity of Tregs in our patients. We previously reported that Tregs are increased in metastatic RCC patients but decreased to normal levels in those patients responding to IL-2 therapy [15]. In the current study, we similarly found that the Treg population was increased in patients compared to normal donors without detectable differences in suppressor activity. Patients who achieved stable disease demonstrated a 50% reduction in the mean number of Tregs within four weeks of completing the first course of IL-2 (p = 0.006) and supports the notion that patients destined to respond to immunotherapy exhibit a decreased frequency of Tregs. In murine tumor models, the ratio of effector to regulatory T cells was found to be the critical determinant of tumor regression or progression [28]. Similarly, we found that patients with stable disease exhibited an increase in the effector to regulatory ratio that persisted for at least 24 months; in contrast, patients with progressive disease showed a low ratio at all time points tested. Although we lacked statistical power in our trial to directly compare these groups, these data would support determining the effector to regulatory ratio in future clinical trials.

In summary, this study provides safety and feasibility data supporting the combination of MVA-5T4 vaccine and IL-2 for patients with metastatic RCC. The treatment regimen was associated with induction of 5T4-specific humoral and cellular immunity. Twelve patients had stable disease, which was associated with increased effector T cells, reduced Tregs and increased effector to regulatory T cell ratios, suggesting a benefit from therapy. Although there was insufficient power to make conclusions regarding clinical response, these data suggest that stable disease by current RECIST criteria might harbor subsets of patients who may benefit from immunotherapy. Future randomized studies will be helpful in better delineating the potential effectiveness of MVA-5T4 and IL-2 for the treatment of RCC.

## Competing interests

Richard Harrop, William Shingler and Stuart Naylor are employed by Oxford Biomedica U.K. Ltd.

## Authors' contributions

H. L. K and M.W.C. did the conception and design of the clinical study; H. L. K., B. T and W. S. treated and evaluated patients; G. D. and J. M. provided study materials; S. K-S, D. W. K, W. H. S, D. M. processed samples and analyzed immune responses; H.L.K, S. K-S, J. N. H, R. H., and S. N. did data analysis and interpretation. H.L.K, J. N. H and S. K-S did statistical analysis and wrote the manuscript. All authors have agreed to all the content in the manuscript, including the data as presented.

## Supplementary Material

Additional file 1 **MVA- and 5T4- specific antibody responses. (A) MVA-specific antibody titers, (B) 5T4-specific antibody titers**. The data provided antibody titers specific for MVA- and 5T4- antibodies. Click here for file
