# Supplementary material for: Phase II trial of Modified Vaccinia Ankara (MVA) virus expressing 5T4 and high dose Interleukin-2 (IL-2) in patients with metastatic renal cell carcinoma
Source: J Transl Med. 2009 Jan 7;7:2. doi: 10.1186/1479-5876-7-2 (PMC2631474; doi:10.1186/1479-5876-7-2)
Supplement: Additional file 1 — MVA- and 5T4- specific antibody responses. (A) MVA-specific antibody titers, (B) 5T4-specific antibody titers. The data provided antibody titers specific for MVA- and 5T4- antibodies. [file 1479-5876-7-2-S1.pdf]

Additional file 1. MVA- and 5T4- specific antibody responses

a. MVA-specific antibody titers

Patients	0 wk	3 wk	6 wk	9 wk	12 wk	15 wk	21 wk	26 wk	29 wk	39 wk	42 wk	52 wk	55 wk	65 wk	78 wk	92 wk	105 wk
1	<1000	**16000**	**16000**														
2	<1000	**4000**	**4000**	**2000**	**8000**												
3	<1000	**16000**	**32000**	**16000**													
5	<1000	**4000**	**16000**	**16000**	**16000**	**16000**	**4000**	**4000**		**4000**	**8000**	**8000**	**32000**	**16000**	**8000**	**8000**	**8000**
6	<1000	**4000**	**8000**	<1000	**4000**	**8000**	**4000**	**4000**	**4000**	**4000**	**4000**	**2000**	**4000**	**2000**	**4000**		
7	<1000	**32000**	**32000**	**16000**	**16000**	**32000**	**8000**	**8000**	**8000**	**8000**	**8000**	**4000**		**4000**	**4000**	**4000**	
8	<1000	**32000**															
9	<1000	**4000**	**16000**	**8000**	**8000**	**8000**	**8000**	**8000**	**16000**	**8000**	**16000**	**16000**	**16000**	**8000**	**8000**	**8000**	**8000**
10	<1000	**16000**	**8000**	**16000**													
11	<1000	**16000**	**8000**	**16000**	**8000**	**16000**	**4000**	**4000**		**4000**							
12	<1000	**128000**	**128000**	**64000**	**64000**	**64000**	**16000**	**16000**									
13	<1000	**32000**	**64000**	**32000**	**32000**	**32000**	**16000**	**16000**		**16000**		**16000**					
14	<1000	**32000**	**32000**														
15	<1000	**2000**	**8000**	**8000**	**8000**	**8000**	**4000**	**2000**	**8000**	<1000	**8000**	**4000**	**64000**	**16000**	**8000**	**2000**	
16	<1000	**16000**	**64000**	**64000**	**32000**	**16000**	**16000**	**8000**									
17	<1000	**8000**	**8000**	**4000**	**4000**	**4000**	**4000**	**4000**	**8000**	**4000**	**8000**	**4000**		**4000**	**2000**		
19	4000	**128000**	**64000**	**32000**	**16000**	**16000**	**16000**	**16000**	**64000**								
20	<1000	**4000**	**8000**	**8000**	**16000**	**16000**											
21	<1000	**4000**	**4000**	**8000**	**8000**												
22	<1000	<1000	**2000**	**2000**													
23	<1000	**32000**	**16000**	**8000**	**16000**	**8000**		**2000**	**32000**								
24	<1000	**4000**	<1000	<1000	<1000	<1000	<1000										
25	<1000	**2000**	**32000**														

Additional file 1. MVA- and 5T4- specific antibody responses

b. 5T4-specific antibody titers

Patients	0 wk	3 wk	6 wk	9 wk	12 wk	15 wk	21 wk	26 wk	29 wk	39 wk	42 wk	52 wk	55 wk	65 wk	78 wk	92 wk	105 wk
1	<10	<10	**160**														
2	<10	**160**	**2560**	**640**	**320**												
3	<10	<10	<10	**10**													
5	<10	<10	**640**	**80**	**20**	**80**	<10	<10		<10	<10	<10	**40**	**10**	<10	<10	<10
6	<10	<10	**80**	**20**	**20**	**20**	**40**	<10	<10	**10**	**20**	<10	**10**	<10	<10		
7	<10	<10	**160**	**80**	**80**	**80**	**20**	**10**	<10	**10**	**160**	**20**		<10	<10	<10	
8	<10	**160**															
9	<10	<10	**2560**	**640**	**160**	**80**	**40**	**20**	**20**	**20**	**20**	<10	<10	<10	<10	<10	<10
10	<10	<10	**320**	**320**													
11	<10	**80**	**640**	**320**	**80**	**80**	**80**	**80**		<10							
12	<10	**160**	**2560**	**1280**	**640**	**320**	**80**	**40**									
13	**40**	**160**	**320**	**160**	**160**	**160**	**80**	**40**		**20**		**40**					
14	<10	**160**	**640**														
15	<10	**80**	**640**	**320**	**80**	**80**	**80**	**40**	**40**	**20**	**80**	**40**	**320**	**10**	**40**	**20**	
16	<10	**160**	**1280**	**1280**	**1280**	**320**	**320**	**80**									
17	<10	<10	**80**	**80**	**80**	<10	<10	<10	<10	<10	<10	<10		<10	<10		
19	<10	<10	**40**	**40**	**20**	<10	<10	<10	<10								
20	<10	<10	**40**	**40**	**40**	**40**											
21	<10	<10	**40**	**40**	**20**												
22	<10	<10	**40**	**40**													
23	**20**	**20**	**40**	**20**	**20**	**20**		<10	**40**								
24	<10	**80**	**40**	**40**	**20**	**40**	**10**	<10									
25	<10	<10	**10**														
